# Arsenic Trioxide Treatment during Pregnancy for Acute Promyelocytic Leukemia in a 22-Year-Old Woman

**DOI:** 10.1155/2020/3686584

**Published:** 2020-03-11

**Authors:** Claire Cochet, Marion Simonet, Julie Cattin, Jean-Patrick Metz, Ana Berceanu, Eric Deconinck, Etienne Daguindau, Françoise Schillinger, Pierre Fenaux, Nicolas Mottet, Yohan Desbrosses

**Affiliations:** ^1^University Hospital of Besancon, Department of Obstetrics, Besançon F-25000, France; ^2^University Hospital of Besancon, Department of Hematology, Besançon F-25000, France; ^3^University Bourgogne Franche-Comté, INSERM, EFS BFC, UMR 1098, Interactions Hôte-Greffon Tumeur/Ingénierie Cellulaire et Génique, Besançon F-25000, France; ^4^Etablissement Français du Sang Bourgogne Franche Comté, Laboratoire d'hématologie-Immunologie-Biologie Moléculaire, Besançon F-25020, France; ^5^Saint Louis Hospital, Paris Diderot University, Paris F-75010, France

## Abstract

Acute leukemia during pregnancy is rare (1 for 100000 pregnancies). The association of arsenic trioxide (ATO) and all-trans retinoic acid (ATRA) is known as the best therapy in standard-risk acute promyelocytic leukemia (APL). We describe the first case of a pregnancy with ATRA and ATO reported in the literature. In March 2018 at the University Hospital of Besançon, a 22-year-old woman was diagnosed with APL at 14 weeks of gestation (WG). She received a total of 2160 mg of ATRA and 930 mg of ATO between 14 and 35 WG. The mother's cytological remission was very fast. No maternal or fetal complications occurred during pregnancy. The pediatrics outcomes were good. Many case reports about ATRA exposure during the second and third trimesters report no serious adverse effect for pregnancy. ATO is teratogenic, genotoxic, and carcinogenic and passes through the placenta. Fetal exposure seems to be associated with bad pregnancy outcomes (preterm delivery, decreased birth weight, and fetal loss) and with lung diseases in young adults. No clinical trial is obviously possible, and the only data available are environmental exposure or animal studies. This case report may help medical teams to make hard decision for a treatment of APL during pregnancy.

## 1. Introduction

Acute promyelocytic leukemia (APL) is a sub-type of acute myeloid leukemia. It is characterized by a translocation between chromosomes 15 and 17 t(15:17), which leads to the fusion between the promyelocytic leukemia protein (PML) gene and the retinoic acid receptor *α* (RAR*α*). The association of arsenic trioxide (ATO) and all-trans retinoic acid (ATRA) is known as the best therapy in standard-risk APL compared to the combination of ATRA and anthracycline-based chemotherapy [1–3].

Acute leukemia during pregnancy is rare (1 for 100000 pregnancies) [4]. APL is curable, but it can have dramatical issues for the mother and the fetus. Its treatment is an emergency. It is associated with ethical questions about the pregnancy outcomes since arsenic is known to be teratogenic. Almost 30 cases of pregnancies with ATRA during the 2^nd^ and/or 3^rd^ trimester are reported, but none with ATO according to the French teratogen agents reference center (CRAT). We describe the case of a treatment of APL in pregnancy with ATRA and ATO in the University Hospital of Besançon.

## 2. Case Description

**Figure 1:**
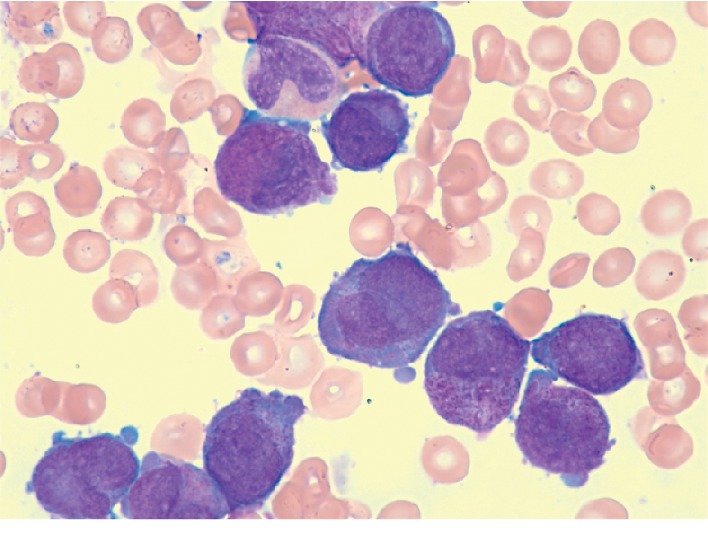
Medullary smear: presence of 82% of hypergranular blasts with bundles of Auer rods.

In March 2018, the patient, a 22-year-old woman with no medical history, was 14 weeks of gestation (WG). She was diagnosed with APL on a blood test, revealing a pancytopenia and a minimal diffuse intravascular coagulopathy (white blood cells (WBC) count 4.2 × 10^9^/L with 6% of circulating hypergranular blasts with bilobed nuclear, neutrophil cells count 2 × 10^9^/L, hemoglobin rate 11.6 g/dL, and platelet count 8 × 10^9^/L). Bone marrow aspiration confirmed the diagnosis: 82% of hypergranular blasts with bundles of Auer rods and a translocation t(15;17) on cytogenetic analyses (Figure 1). The ratio PML-RAR*α* was 0.47.

ATRA (45 mg/m^2^/day per os) was introduced immediately after the APL suspicion. The couple was informed about the unknown consequences of the recommended treatment for APL and the absence of malformation risk after 10 WG. There was no pregnancy termination demand.

**Figure 2:**
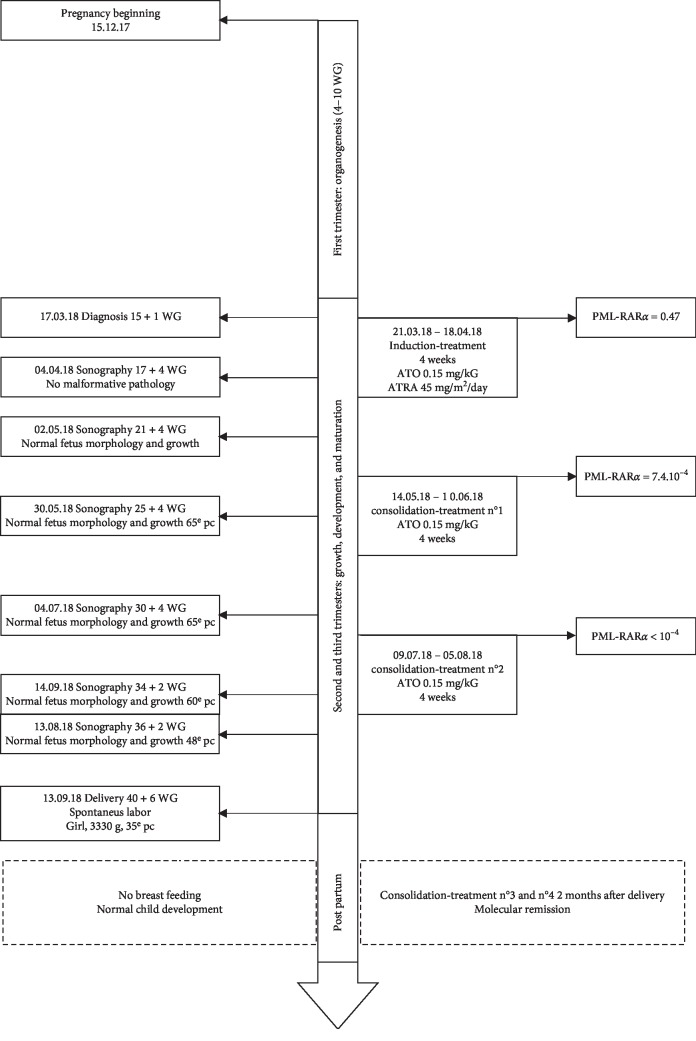
Chronology of treatment received, assessment of disease response, and pregnancy control.

We started a complete induction-treatment and added ATO 0.15 mg/kg/day with ATRA (45 mg/m^2^/day) until the hematological complete remission (Figure 2). Dexamethasone 10 mg twice daily was administered to prevent ATRA differentiation syndrome. Heparin (50 UI/kg) was also introduced to prevent thrombotic events. The induction-treatment ended after 4 weeks. The tolerance was good. We quickly obtained a complete cytological remission with minimal residual disease (MRD) (PML-RAR*α* = 7.4×10^−4^).

The consolidation-treatment consisted in ATRA (45 mg/m^2^/day) 7 cycles of 2 weeks each 4 weeks and 4 cycles of ATO of 4 weeks each 8 weeks. The MRD became undetectable after the first consolidation-treatment's cycle.

Pregnancy monitoring consisted in monthly ultrasound scans and medical examinations. Sonographies showed no fetal abnormality especially concerning neurological, renal, and cardiac development. Fetus vitality was normal, and its growth reached the 50^th^ percentile. No maternal complication occurred: no bleeding, tension disorder, or preterm birth threat. Clinical examinations and biological observations showed no particularity. No additional treatment was necessary. The hemoglobin rate remained >10 g/dL during the pregnancy.

Since the patient's biological response was total, the consolidation-treatment was suspended after 2 cycles (around 35 WG) in anticipation of the delivery, to avoid perinatal fetal exposure of arsenic. Pregnancy progress was still totally normal, and no induction of labor was necessary.

At 40 + 6 WG, our patient gave birth to a healthy girl of 3330 g with an Apgar score of 10 at 5 minutes. The delivery process was normal. The blood sample was proceeded on the new born cord to monitor the hepatic and renal functions. Two electrocardiograms and two blood samples at day 1 and day 4 did not show any cardiac or electrolytic complication. Breast feeding was forbidden.

The consolidation-treatment was resumed 2 months after the delivery.

After the complete treatment, the cytological remission was complete with an undetectable MRD. The patient confessed she did not follow the medical prescription and never took the ATRA during the prenatal consolidation-treatment phase. The patient received a total of 2160 mg of ATRA and 930 mg of ATO while she was pregnant.

The pediatric control 3 months and 9 months after birth showed no complication. The baby's growth and development were normal.

## 3. Results and Discussion

Cancers during pregnancy represent clinical and ethical dilemmas for medical teams. So far, evidences on pediatric outcomes after most of maternal cancer's treatment during pregnancy are reassuring [5]. APL is an oncologic emergency known to increase the risk of abortion, perinatal mortality, preterm delivery, and intrauterine growth restriction during pregnancy [6].

The combination of ATO and ATRA without chemotherapy significantly improved survival and relapse risk in patients with newly diagnosed standard-risk APL [1–3]. The French-Belgian-Swiss APL group proved in APL 2006 trial that ATO, added to a “classical” ATRA and anthracycline-based chemotherapy regimen, reduces the incidence of relapse in standard-risk APL [7]. Since the superiority of this treatment is proved, medical teams will have to deal with the question of arsenic use in pregnant women with APL. Our case confirms ATO's relevance since the patient confessed she did not take her ATRA-based treatment during the consolidation phase.

ATRA is teratogenic when given during organogenesis. When used during the 2^nd^ or 3^rd^ trimester of pregnancy in human, fetal arrhythmia and retarded-fetal growth have been described [8]. In our case, there was no teratogenic risk since the treatment began after the organogenesis period (4–10 WG). Many cases of pregnancies with ATRA during the second and third trimesters are reported in the literature with no serious adverse outcomes [6, 9–11].

ATO is teratogenic, genotoxic, and carcinogenic. No case report on ATO's use during pregnancy in human are available in the literature, but it passes through the placenta [12]. Animal studies proved the carcinogenic effect of high doses of arsenic (up to 10 mg/kG/day) [12, 13]. There is a variation of sensibility to arsenic exposure due to the rate of methylation, which is very different between species. Human sensibility is still unknown [14]. The only data available on intrauterine arsenic exposure effects are environmental studies. It seems to be associated with malignant and nonmalignant lung diseases in young adults through epigenetic effects [15]. Studies in Bangladesh and Taiwan showed that arsenic environmental fetal exposure is associated with decreased birth weight and preterm delivery [16, 17]. A Bangladesh cohort study also showed that there is a bigger risk of fetal loss and infant death for women who drink water with more than 50 microgram of arsenic per liter. It also seems to be a dose effect [16]. All those studies are observational and retrospective. The exact dose of arsenic maternal exposure is difficult to weight. Moreover, the environmental exposure also affects the periconceptional period and first trimester of pregnancy. In our case, the patient received more than 900 mg of arsenic trioxide. It is far more than in environmental studies. However, this exposure was limited to the second and third trimesters of pregnancy with a therapeutic window for the delivery. Since no clinical trials are obviously possible on pregnant women, the generalization of our case is difficult.

Regarding the treatment choice, it is important to highlight that any delay can compromise the remission in APL. At time of diagnosis, a medical termination of pregnancy was impossible because our patient had cytopenia and a minimal diffuse intravascular coagulopathy. Despite the limited clinical experience, ATRA was introduced immediately. This treatment seemed reasonably safe when used in patients with APL in second and third trimesters of pregnancy. Indeed, recommendations from the European LeukemiaNet published in 2009 recommend the use of ATRA and chemotherapy in these patients and an induction of labor between cycles of chemotherapy [18]. The update of these recommendations in 2019 did not make any change in the management of APL during pregnancy [19]. Anthracycline-based chemotherapy can increase the risk of abortion, prematurity, low weight, neonatal neutropenia, and sepsis [18]. For these reasons, we were reluctant to use chemotherapy during pregnancy. Moreover, anthracycline-based chemotherapy induces a long aplasia which can cause infections and hemorrhagic complications. The expected response rate with ATRA alone is not significantly different from ATRA plus chemotherapy in terms of complete remission rate, but it can have unfavorable impact on the risk of relapse [18]. Therefore, after a multidisciplinary consultation, we decided to use ATRA and ATO treatment with stringent fetal monitoring.

In our case, no adverse fetal outcome occurred. The mother's response was very fast, and it seems that pregnancy did not have effect on treatment efficiency. It will take time to assess the long-term effect on the child development of prenatal ATO exposure. Nevertheless, this unique case report may help medical teams to make hard decision for treatment of acute promyelocytic leukemia during pregnancy, whose best option is nowadays known to be ATRA and ATO.
